# Oncogene-Expressing Senescent Melanocytes Up-Regulate MHC Class II, a Candidate Melanoma Suppressor Function

**DOI:** 10.1016/j.jid.2017.05.030

**Published:** 2017-10

**Authors:** John van Tuyn, Farah Jaber-Hijazi, Douglas MacKenzie, John J. Cole, Elizabeth Mann, Jeff S. Pawlikowski, Taranjit Singh Rai, David M. Nelson, Tony McBryan, Andre Ivanov, Karen Blyth, Hong Wu, Simon Milling, Peter D. Adams

**Affiliations:** 1 Institute of Cancer Sciences, University of Glasgow, Garscube Estate, Switchback Road, Glasgow, UK; 2 Institute for Infection, Immunity and Inflammation, University of Glasgow, Glasgow, UK; 3 Vanderbilt University Medical Center, Nashville, Tennessee, USA; 4 Institute of Biomedical and Environmental Health Research, University of the West of Scotland, Paisley, UK; 5 Barts Cancer Institute. Queen Mary, University of London. Charterhouse Square, London, UK; 6 Beatson Institute for Cancer Research, Garscube Estate. Switchback Road, Glasgow, UK; 7 Fox Chase Cancer Center, Philadelphia, Pennsylvania, USA; 8 Sanford Burnham Prebys Medical Discovery Institute, San Diego, California, USA

**Keywords:** MHC, major histocompatibility complex, NK, natural killer, OIS, oncogene-induced senescence, RNA-seq, RNA sequencing, SA β-gal, senescence-associated β-galactosidase

## Abstract

On acquisition of an oncogenic mutation, primary human and mouse cells can enter oncogene-induced senescence (OIS). OIS is characterized by a stable proliferation arrest and secretion of proinflammatory cytokines and chemokines, the senescence-associated secretory phenotype. Proliferation arrest and the senescence-associated secretory phenotype collaborate to enact tumor suppression, the former by blocking cell proliferation and the latter by recruiting immune cells to clear damaged cells. However, the interactions of OIS cells with the immune system are still poorly defined. Here, we show that engagement of OIS in primary human melanocytes, specifically by melanoma driver mutations *NRASQ61K* and *BRAFV600E*, causes expression of the major histocompatibility class II antigen presentation apparatus, via secreted IL-1ß signaling and expression of CIITA, a master regulator of major histocompatibility class II gene transcription. In vitro, OIS melanocytes activate T-cell proliferation. In vivo, nonproliferating oncogene-expressing melanocytes localize to skin-draining lymph nodes, where they induce T-cell proliferation and an antigen presentation gene expression signature. In patients, expression of major histocompatibility class II in melanoma is linked to favorable disease outcome. We propose that OIS in melanocytes is accompanied by an antigen presentation phenotype, likely to promote tumor suppression via activation of the adaptive immune system.

## Introduction

Melanoma is a frequently fatal cancer originating from pigment-producing melanocytes of the skin (Lo and Fisher, 2014). The most common mutations found in melanoma are those that activate the mitogen-activated protein kinase signaling pathway, most notably in *BRAF* and *NRAS* (Lo and Fisher, 2014). The same mutations are also commonly found in benign nevi (or moles) (Omholt et al., 2002, Pollock et al., 2003). However, benign nevi only rarely progress to cancer because oncogene-expressing nevus melanocytes are ultimately checked in a proliferation-arrested state called *oncogene-induced senescence* (OIS) (Munoz-Espin and Serrano, 2014). Nevus melanocytes express several molecular markers of senescence, including senescence-associated ß-galactosidase (SA ß-gal) and tumor suppressor p16INK4a (Gray-Schopfer et al., 2006, Michaloglou et al., 2005, Pawlikowski et al., 2013, Suram et al., 2012). Aggregates of apparently nonmalignant, nonproliferative, p16INK4a-expressing, melanocytic nevus-like cells, in the absence of any concurrent or subsequent melanoma, have also been well documented in the skin-draining lymph nodes of humans (Mihic-Probst et al., 2003, Patterson, 2004).

OIS is also characterized by a secretory program, the senescence-associated secretory phenotype (Acosta et al., 2008, Krtolica et al., 2001, Kuilman et al., 2008). The senescence-associated secretory phenotype has various functions in OIS, including reinforcement and maintenance of proliferation arrest (Acosta et al., 2008, Kuilman et al., 2008) and recruitment of macrophages, neutrophils, and natural killer (NK) cells of the innate immune system to clear premalignant oncogene-expressing senescent cells (Xue et al., 2007). NK cells also clear senescent cells in response to other cell- and tissue-damaging stresses (Krizhanovsky et al., 2008, Soriani et al., 2009). However, clearance of mouse hepatocytes expressing an activated *NrasQ61K* oncogene was also shown to depend on activation of adaptive immunity, specifically on CD4^+^ T cells (Kang et al., 2011). Typically, CD4+ T cells are activated in the secondary lymphoid tissues, such as lymph nodes and spleen, by professional antigen-presenting cells such as dendritic cells (Trombetta and Mellman, 2005). Dendritic cells endocytose and process antigens in peripheral tissues and then migrate via the lymphatic vessels to the lymph nodes, where they activate CD4^+^ T cells by major histocompatibility (MHC) class II-mediated antigen presentation to naïve T cells.

How senescent cells can activate the adaptive immune systems has been a mystery. Here we show that OIS in melanocytes caused by activation of the RAS/mitogen-activated protein kinase pathway is accompanied by dramatic up-regulation of the MHC class II antigen presentation complex. Furthermore, melanocytes carrying either *BrafV600E* or *NrasQ61K* mutations re-localize specifically to skin-draining lymph nodes in mouse models. We also present functional evidence that in the nodes, oncogene-expressing nonproliferating melanocytes enact an antigen presentation function to activate the adaptive immune system.

## Results

### Melanocytes express MHC class II upon oncogene-induced senescence initiated by melanoma driver mutations

**Figure 1:**
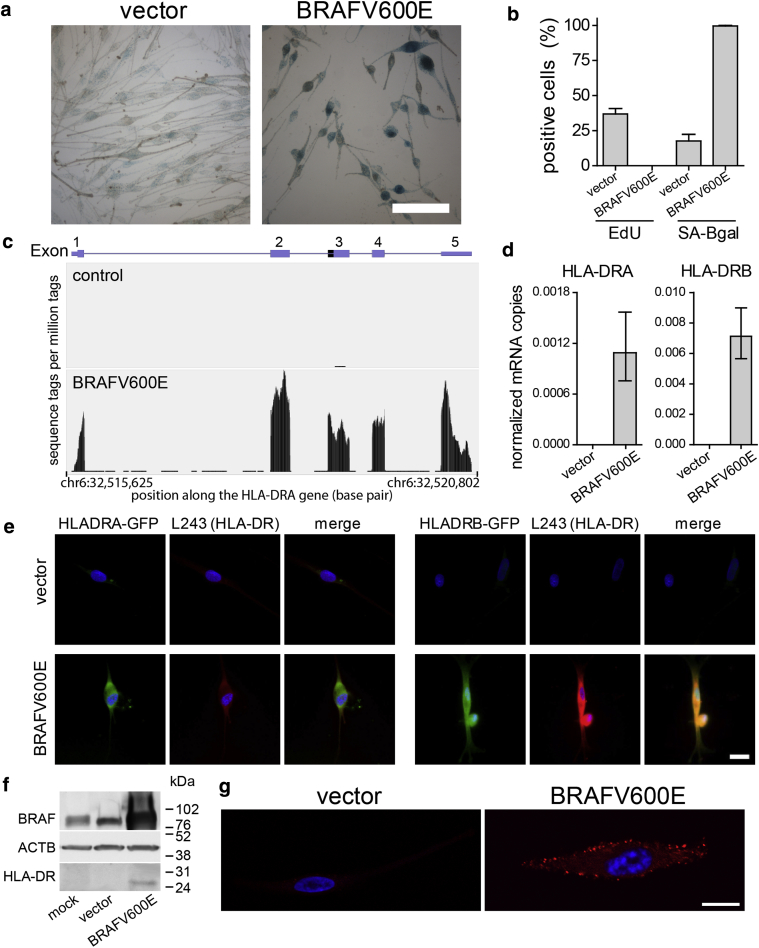
**Melanocytes express MHC class II upon OIS.** (**a**) Staining for SA ß-gal activity (blue) on vector- or BRAF600E-transduced melanocytes. Scale bar = 100 μm. The time span of oncogene activation was kept at 2 weeks for all OIS experiments unless indicated otherwise. (**b**) Quantification of EdU incorporation and SA ß-gal staining. Graph shows means ± standard deviation, n = 3. (**c**) Representative RNA sequencing track showing HLA-DRA sequence reads for control and BRAFV600E-transduced melanocytes. Y-axis shows sequence tags per million tags; x-axis shows position along the HLA-DRA gene (spanning 5,178 base pairs), with boxed exons. (**d**) Detection of HLA-DRA transcript levels by quantitative real-time reverse transcriptase–PCR analysis. (**e**) Representative immunofluorescent image of HLA-DRA-GFP (left three panels) and HLA-DRB-GFP (right three panels) fusion protein-transduced melanocytes. GFP staining in green, staining for MHC class II in red, and nuclei in blue. Scale bar = 200 μm. (**f**) Western blot showing BRAF, beta-actin (ATCB), and MHC class II (HLA-DR) expression. (**g**) Confocal immunofluorescent image of melanocytes stained for MHC class II (HLA-DR; red) and nuclei (blue). Scale bar = 10 μm. All graphs show means ± standard deviation, n = 4. chr, chromosome; MHC, major histocompatibility; OIS, oncogene-induced senescence; SA-Bgal, senescence-associated β-galactosidase.

As we and others previously showed (Michaloglou et al., 2005, Pawlikowski et al., 2013), ectopic expression of BRAFV600E in primary human melanocytes induces OIS. Indicative of senescence, BRAFV600E-expressing melanocytes up-regulated SA ß-gal (Figure 1a, 1b), arrested DNA replication as determined by a lack of EdU incorporation (Figure 1b) and showed senescence-associated heterochromatin foci in the nucleus (see Supplementary Figure S1 online).

We previously investigated the transcriptional changes of melanocytes undergoing OIS by whole-genome microarray analysis and RNA sequencing (RNA-seq) analysis (Pawlikowski et al., 2013). Comparing BRAFV600E-expressing melanocytes against vector-transduced and uninfected melanocytes, we observed striking up-regulation of MHC class II complex transcripts (Figure 1c, Supplementary Table S1 online). The cell surface of MHC class II complex is a heterodimer of α and ß polypeptides, for example HLA-DRA and HLA-DRB1, respectively. Antigen presentation also depends on expression of chaperones and accessory factors, such as HLA-DM, HLA-D0, and CD74, which facilitate antigen processing, loading, and presentation by the MHC class II complex (Trombetta and Mellman, 2005). We observed HLA-DRA and HLA-DRB among the top changes (see Supplementary Table S1). In addition, related HLA-DQA, HLA-DQB, HLA-DPA, and HLA-DPB were also up-regulated, together with important antigen processing and presentation accessory molecules, such as HLA-DMA, HLA-DMB, and invariant chain CD74 (see Supplementary Tables S2, S3 online).

Up-regulation of the major components of MHC class II mRNAs HLA-DRA and HLA-DRB was confirmed by quantitative real-time reverse transcriptase–PCR (Figure 1d). Expression of BRAFV600E also enhanced expression of ectopic HLA-DRA and HLA-DRB GFP fusion proteins (Figure 1e). Because this increase is independent of the genes’ normal transcription control elements, this also suggests at least some level of posttranscriptional regulation. Expression of endogenous HLA-DR protein in melanocytes upon OIS was shown by Western blot analysis (Figure 1f) and immunofluorescence (Figure 1g, Supplementary Figure S2 online). A significant fraction of HLA-DR was localized at the plasma membrane of senescent cells (Figure 1g, Supplementary Figure S2b), consistent with a role in antigen presentation.

**Figure 2:**
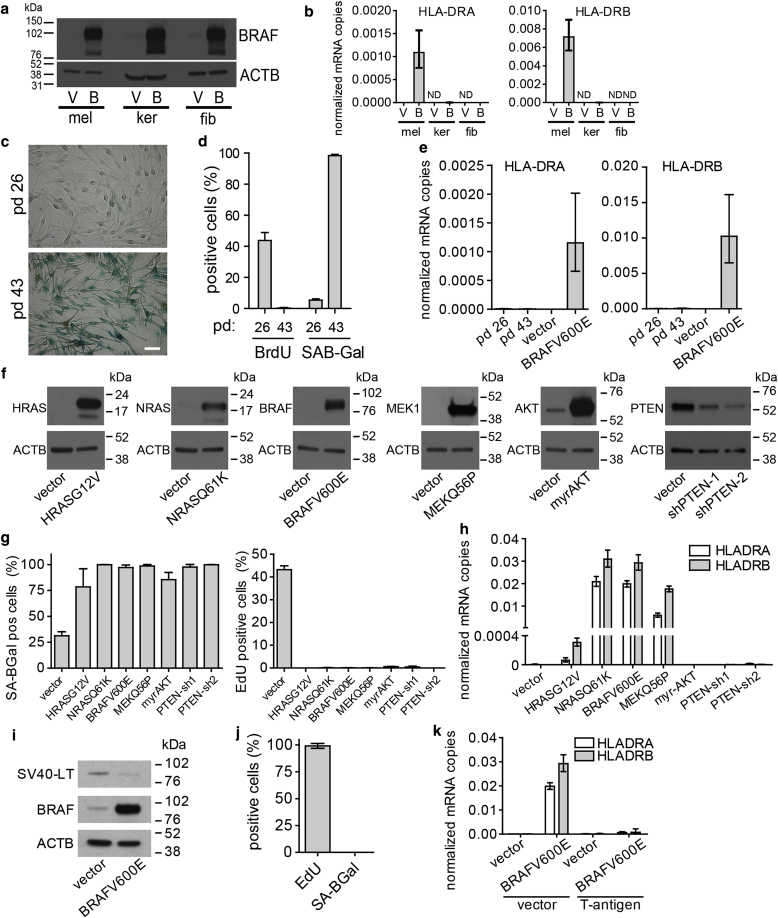
**Expression of MHC class II is specific to melanocytes and OIS.** (**a**) Western blot of BRAFV600E expression in melanocytes, keratinocytes, and fibroblasts transduced with a vector encoding BRAFV600E (B) or control vector (V). (**b**) HLA-DRA and HLA-DRB transcript levels detected by quantitative real-time reverse transcriptase–PCR analysis, in melanocytes (mel), fibroblasts (fib), or keratinocytes (ker). ND indicates no signal detected. (**c**) Staining for SA ß-gal activity in melanocytes in exponential growth phase (PD 26) or at replicative senescence (PD 43). Scale bar = 50 μm. (**d**) Quantification of SA ß-gal– and BrdU-positive melanocytes. (**e**) HLA-DRA and HLA-DRB transcript levels in melanocytes detected by quantitative real-time reverse transcriptase–PCR. (**f**) Western blots of HRASG12V, NRASQ61K, BRAFV600E, MEKQ56P, and myrAKT expression and knockdown of PTEN. (**g**) Quantification of SA ß-gal staining (left) and EdU incorporation (right) of melanocytes transduced as indicated. (**h**) HLA-DRA and HLA-DRB transcript levels detected by quantitative real-time reverse transcriptase–PCR of melanocytes transduced as indicated. (**i**) Western blot showing expression of BRAFV600E and SV40 T antigen. (**j**) Quantification of SA ß-gal staining and EdU incorporation of melanocytes transduced with BRAFV600E and SV40 T-antigen. (**k**) All graphs show means ± standard deviation, n = 4. MHC, major histocompatibility complex; SA ß-gal, senescence-associated β-galactosidase; SA-Bgal, senescence-associated β-galactosidase.

To establish whether BRAFV600E-associated MHC class II expression is cell-type restricted, we transduced melanocytes, primary human fibroblasts (IMR90), and primary human epidermal keratinocytes with BRAFV600E or control vector (Figure 2Aa). In contrast to BRAFV600E-transduced melanocytes, neither fibroblasts nor keratinocytes exhibited significant up-regulation of MHC class II transcript levels (Figure 2b). Furthermore, gene expression profiling of fibroblasts transduced with BRAFV600E showed marked down-regulation of proliferation-promoting genes and up-regulation of inflammatory/senescence-associated secretory phenotype genes characteristic of senescence (see Supplementary Figure S3 online) and confirmed no significantly up-regulated MHC class II genes (see Supplementary Figure S4 online). These results together suggest that MHC class II induction is not a common feature of all primary cell types in response to OIS (fibroblasts) and/or oncogene expression (keratinocytes).

In addition to activated oncogenes, other triggers also initiate cell senescence. For example, so-called *replicative senescence,* which results from excess rounds of cell division (Salama et al., 2014). To test whether MHC class II up-regulation is common to different modes of senescence, we investigated replicative senescence melanocytes. Melanocytes were serially passaged until they ceased proliferation and were confirmed to show replicative senescence by positive staining for SA ß-gal (Figure 2c, 2d) and lack of BrdU incorporation (Figure 2d). In contrast to BRAFV600E-mediated OIS, replicative senescence did not induce robust up-regulation of HLA-DRA or HLA-DRB, as detected by quantitative real-time reverse transcriptase–PCR (Figure 2e). Finally, no MHC class II protein could be detected by Western blotting (data not shown). These findings suggest that MHC class II induction is specific to OIS in some cell types and not replicative senescence.

To establish whether MHC class II up-regulation in melanocytes is specific to activated BRAFV600E or also triggered by other perturbations of melanoma oncogenic and tumor suppressor pathways, melanocytes were transduced with activated oncogenes, HRASG12V and NRASQ61K, activated MEK1Q56P (Emery et al., 2009), and activated myrAKT, as well as two short hairpin RNAs to stably knock down PTEN (Figure 2f). Ectopic expression of each of these oncogenes and knockdown of PTEN resulted in senescence as determined by positive staining for SA ß-gal (Figure 2g, left, and see Supplementary Figure S5 online) and lack of proliferation indicated by the absence of EdU incorporation (Figure 2g, right). Expression of NRASQ61K, BRAFV600E, and MEK1Q56P resulted in robust up-regulation of both HLA-DRA and HLA-DRB, whereas expression of HRASG12V gave rise to a significantly weaker up-regulation of both. In marked contrast, myrAKT expression and PTEN knockdown did not result in detectable HLA-DR up-regulation (Figure 2h). In contrast to results reported here, PTEN knockdown has previously been reported to not cause induction of senescence (Vredeveld et al., 2012). There could be many reasons for this apparent discrepancy—genetic and/or epigenetic differences in the melanocytes, cell culture conditions, and others. Regardless, these results suggest that, at least under conditions used here, MHC class II induction is specific to aberrant mitogenic signaling through the RAS/BRAF/ERK pathway but not the PTEN-Akt signaling pathway.

Because MHC class II induction in BRAFV600E mutant melanocytes occurs concomitantly with induction of senescence, we wished to elucidate whether MHC class II induction is dependent on known effectors of the senescence program. In human cells, an intact p53 or pRB tumor suppressor pathway is necessary for initiation and maintenance of the senescence program (Salama et al., 2014), and abolition of p53/pRB signaling by ectopic expression of SV40 T-antigen disrupts most, if not all, hallmarks of senescence (Shay et al., 1991). To test whether MHC class II induction is abolished by ectopic expression of SV40 T antigen, we co-expressed BRAFV600E together with SV40 T antigen (Figure 2i). As expected, T-antigen prevented the growth arrest and SA ß-gal staining associated with senescence in BRAF-expressing melanocytes (Figure 2j, Supplementary Figure S6 online). Moreover, co-expression of SV40 T antigen abolished virtually all of the MHC class II induction (Figure 2k). This suggests that MHC class II induction is not only initiated by expression of oncogenic BRAFV600E but requires at least some elements of the p53/pRB-dependent senescence effector pathways.

### MHC class II up-regulation is mediated by an IL-1ß–CIITA signaling loop

**Figure 3:**
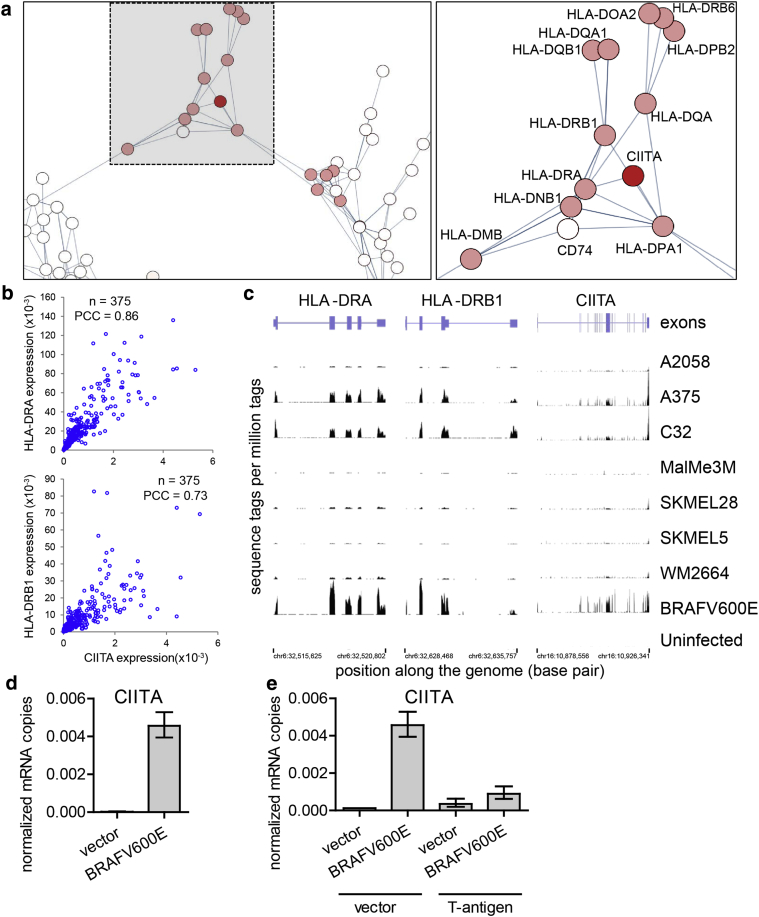
**CIITA is a candidate regulator of MHC class II in OIS melanocytes.** (**a**) Whole transcriptome expression correlation network centered on CIITA generated from The Cancer Genome Atlas melanoma RNA sequencing data. Genes are represented as nodes and a Pearson Correlation Coefficient (PCC) between two genes of at least 0.6 as an edge (n = 375). CIITA is shaded dark red, and HLA genes are light red. The boxed highlighted region in the left panel is shown in the right panel. (**b**) Scatterplots of The Cancer Genome Atlas melanoma RNA sequencing data comparing the expression of CIITA versus HLA-DRA (left) and CIITA versus HLA-DRB1 (right) in 375 patients. (**c**) Representative University of California Santa Cruz (UCSC) genome browser tracks of library normalized RNA sequencing expression at HLA-DRA, HLA-DRB1, and CIITA in seven melanoma cell lines, melanocytes infected with BRAFV600E, and uninfected melanocytes. Y-axis shows sequence tags per million tags; x-axis shows position along the HLA-DRA gene, with boxed exons. (**d**) Quantitative real-time reverse transcriptase–PCR analysis of CIITA transcript levels detected in melanocytes. Graph depicts means ± standard deviation, n = 4. (**e**) CIITA transcript levels detected by quantitative real-time reverse transcriptase–PCR. Graph depicts means ± standard deviation, n = 4. MHC, major histocompatibility complex; OIS, oncogene-induced senescence.

Even though expression of SV40 T antigen suppressed induction of HLA-DRA and HLA-DRB (Figure 2i–k), it is well documented that MHC class II expression is typically found in 50–60% of freshly isolated melanomas (Taramelli et al., 1986). We exploited this observation from the melanocytic lineage to gain insight into candidate regulators of MHC class II in primary human melanocytes. We assessed the correlation between mRNA expression of HLA-DRA and HLA-DRB and all other genes in the publicly available skin cutaneous melanoma gene expression datasets from the Cancer Genome Atlas (TCGA; http://cancergenome.nih.gov/). This showed that, across all these datasets, expression of both HLA-DRA and HLA-DRB correlated most strongly with other HLA molecules (e.g., HLA-DQ and HLA-DP); other molecules involved in antigen presentation (e.g., CD74); and CIITA, a transcription factor already known to drive expression of HLA-DRA and HLA-DRB in dendritic cells (Figure 3a, 3b) (Muhlethaler-Mottet et al., 1997). Conversely, expression of CIITA correlated most strongly with expression of MHC class II antigen presentation molecules (Figure 3a, 3b). To confirm this correlation, we analyzed RNA-seq data from seven different melanoma-derived cell lines (Pawlikowski et al., 2013) and found that CIITA transcript levels correlated strongly with HLA-DRA and HLA-DRB levels across this panel of melanoma lines (Figure 3c). Moreover, we found that MHC class II induction in melanocytes upon BRAFV600E-mediated OIS is also accompanied by increased CIITA expression (Figure 3d). Like expression of MHC class II, oncogene-induced expression of CIITA was abolished by SV40 T-antigen (Figure 3e). Consistent with up-regulation of MHC class II in OIS melanocytes, but not other modes of senescence (Figure 2), we did not observe up-regulation of CIITA in OIS fibroblasts (see Supplementary Figure S4). Together, these data from melanoma tumors, cell lines, and primary human melanocytes indicate that CIITA is a likely driver of HLA-DRA and HLA-DRB in OIS primary human melanocytes.

**Figure 4:**
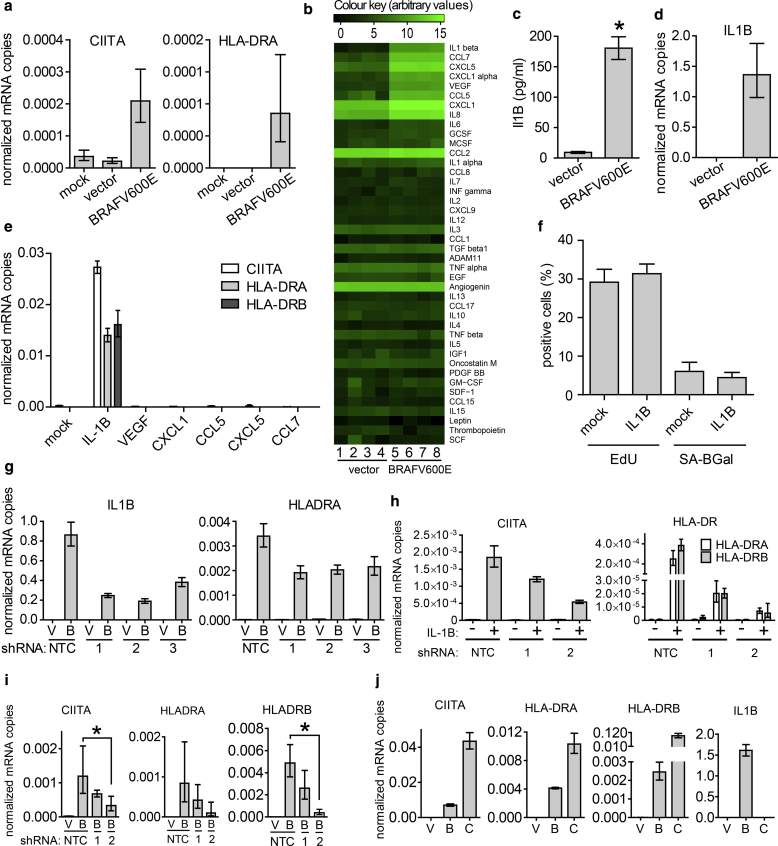
**Expression of MHC class II is controlled by an IL-1ß–CIITA loop.** (**a**) qRT-PCR analysis of CIITA and HLA-DRA transcripts in melanocytes exposed to conditioned medium for 14 days from mock, vector, and BRAFV600E-transduced melanocytes. Graphs depict means ± standard deviation, n = 4. (**b**) Heat map of cytokines in conditioned culture medium. See Supplementary Table S4 for fold change and significance in cytokine levels. (**c**) Quantification by ELISA of IL-1ß in conditioned culture medium. Graph shows mean ± standard deviation, n = 4. ^∗^*P* < 0.0001. (**d**) qRT-PCR analysis of IL-1ß transcripts. Graphs depict means ± standard deviation, n = 4. (**e**) CIITA, HLA-DRA, and HLA-DRB transcripts detected by qRT-PCR of mock or cytokine-treated melanocytes. Cells were cultured for 6 days in culture medium supplemented with 10 ng/ml of the indicated cytokine. Graph depicts means ± standard deviation, n = 3. (**f**) Quantification of EdU incorporation and SA ß-gal staining of melanocytes. Graphs are means ± standard deviation, n = 4. (**g**) qRT-PCR analysis of IL-1ß and HLA-DRA transcripts in melanocytes transduced with vector (V) or BRAFV600E (B) in combination with an shRNA to IL-1ß or nontargeting control (NTC). Graphs depict means ± standard deviation, n = 4. (**h**) qRT-PCR analysis of CIITA, HLA-DRA, and HLA-DRB transcripts in melanocytes induced with vehicle or recombinant IL-1ß and transduced with shRNAs against CIITA or nontargeting control (NTC). (**i**) qRT-PCR analysis of CIITA, HLA-DRA, and HLA-DRB transcripts in melanocytes transduced with vector or BRAFV600E and an shRNA against CIITA or nontargeting control (NTC). Graphs depict means ± standard deviation, n = 4. ^∗^*P* < 0.05. (**j**) qRT-PCR analysis of CIITA, HLA-DRA, HLA-DRB, and IL-1ß transcript levels in melanocytes transduced with control vector (V), BRAFV600E (B), or CIITA (C) overexpression vectors. Graphs depict means ± standard deviation, n = 4. MHC, major histocompatibility complex; qRT-PCR, quantitative real-time reverse transcriptase–PCR; SA ß-gal, senescence-associated β-galactosidase; shRNA, short hairpin RNA.

In cells known to conditionally express CIITA, expression is frequently induced by extracellular ligands (Trombetta and Mellman, 2005). Significantly, naïve melanocytes exposed to medium from OIS melanocytes up-regulated CIITA transcripts (Figure 4a) in conjunction with MHC class II expression (Figure 4a). To identify the extracellular factors responsible for up-regulation of CIITA and MHC class II expression, we probed conditioned medium with an antibody array. Medium from BRAFV600E OIS melanocytes contained increased amounts of inflammatory cytokines compared with conditioned medium from proliferating cells (Figure 4b, Supplementary Table S4 online), including IL-1ß (in the form of either uncleaved pro-IL1ß or cleaved mature IL-1ß), CCL7, CXCL5, CXCL1, vascular endothelial growth factor, and CCL5. The presence of one or more IL-1ß (180 ± 9 pg/ml, n = 4) isoforms in the extracellular medium of OIS melanocytes was confirmed by ELISA (Figure 4c), and robust up-regulation of IL-1ß mRNA transcripts was detected by quantitative real-time reverse transcriptase–PCR (Figure 4d). When added as recombinant proteins to primary human melanocytes, from a panel of secreted cytokines, only mature IL-1ß was able to induce CIITA and MHC class II expression (Figure 4e), but without accompanying activation of senescence, as evidenced by lack of SA ß-gal staining and unimpeded DNA synthesis (Figure 4f). Conversely, partial knockdown of IL-1ß using three independent short hairpin RNAs (Figure 4g) reduced MHC class II induction by BRAFV600E (Figure 4g). Although we have not formally confirmed secretion of cleaved mature IL-1ß in this study (rather than uncleaved pro–IL-1ß), previous studies have shown that OIS cells do secrete processed mature IL-1ß (Acosta et al., 2013), and IL-1α and IL-1ß are key upstream regulators of the senescence-associated secretory phenotype (Acosta et al., 2013, Orjalo et al., 2009). In sum, consistent with these previous studies, our studies indicate a central role for extracellular IL-1ß in induction of MHC class II.

We next tested whether IL-1ß–mediated up-regulation of MHC class II also depends on CIITA. In support of this idea, stimulation of melanocytes with IFN-γ, a well-known inducer of CIITA (Trombetta and Mellman, 2005), also up-regulated expression of HLA-DRA and HLA-DRB (see Supplementary Figure S7 online). More pointedly, partial knockdown of CIITA using two different short hairpin RNAs (Figure 4h) inhibited HLA-DRA and HLA-DRB expression induced by recombinant IL-1ß (Figure 4k). Knockdown of CIITA with two different short hairpin RNAs also tended to decrease BRAFV600E-induced expression of HLA-DRA and HLA-DRB, and with the most effective short hairpin RNA, the effect on HLA-DRB expression was significant (*P* < 0.05) (Figure 4i). The reduced effectiveness of CIITA knockdown in blocking the effects of activated BRAF compared with IL-1ß suggests that BRAFV600E can act via additional signaling effectors besides CIITA. Consistent with an IL1ß-CIITA-MHC II pathway, ectopic expression of CIITA was sufficient to induce expression of HLA-DRA and HLA-DRB, but not IL-1ß, in melanocytes (Figure 4j). Together, these results indicate that IL-1ß–induced expression of CIITA is a major pathway for expression of MHC class II in OIS melanocytes. BRAFV600E-expressing fibroblasts up-regulate IL-1ß but neither CIITA nor MHC II (see Supplementary Figure S4). This suggests that functional coupling between IL-1ß and CIITA occurs in OIS melanocytes but not fibroblasts.

### Oncogene activation causes localization of melanocytes to lymph nodes and T-cell activation

**Figure 5:**
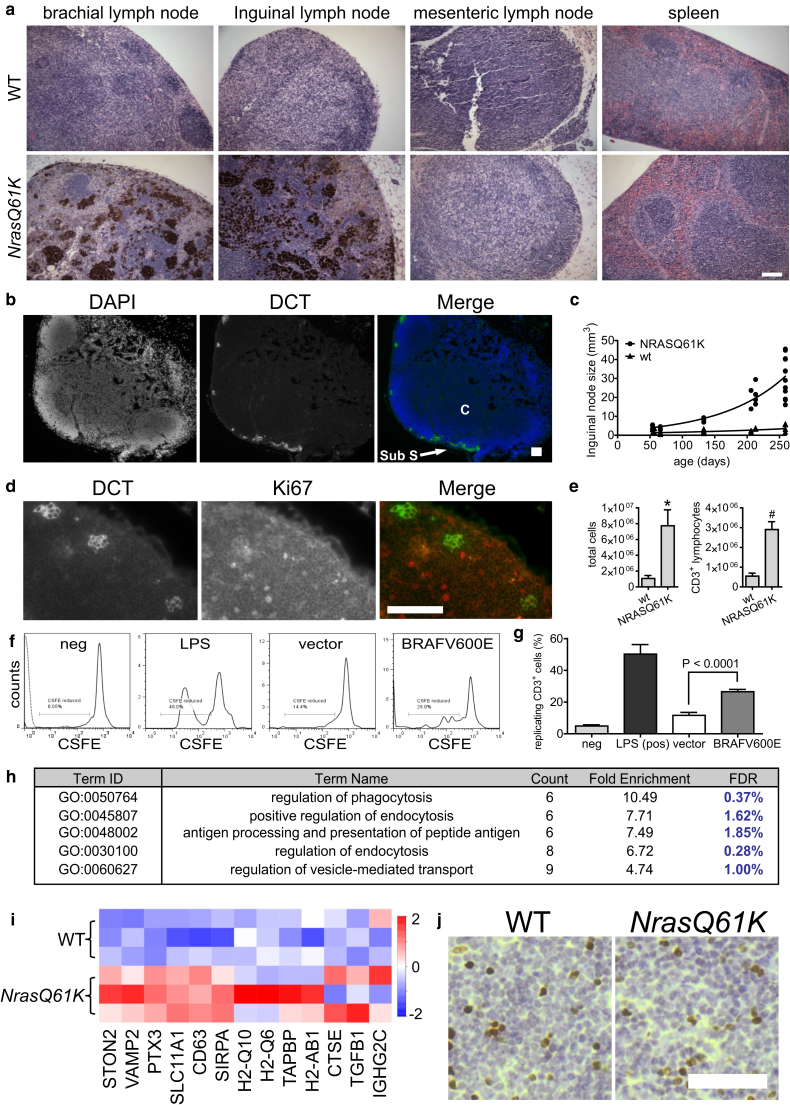
**Oncogene-expressing melanocytes localize to skin-draining lymph nodes.** (**a**) Hematoxylin and eosin-stained sections of lymph nodes and spleen of WT and *Tyr-NrasQ61K* mice. Scale bar = 100 μm. (**b**) DCT (green)-expressing cells in lymph node. DAPI, blue. C, cortex; Sub S, subcapsular sinus. Scale bar = 100 μm. (**c**) Inguinal lymph node size plotted against age of mouse. Each point is a single node (two nodes per mouse). (**d**) Melanocytes detected by DCT (green) in lymph nodes of albino *Tyr-NrasQ61K* transgenic mice and cycling cells detected by Ki67 (red). Scale bar = 100 μm. (**e**) Quantification of total nonmelanocyte (nonpigmented, left) and CD3^+^ fraction (right) from pooled inguinal, brachial, and axillary lymph nodes per mouse. Graph plots means ± standard deviation, n = 3 per group. ^∗^7.2-fold difference, *P* = 0.0049. ^#^5.3-fold difference, *P* = 0.0007. (**f**) FACS analysis of CSFE levels in CD3^+^ cells after co-culture with WBCs (neg, solid line), WBCs incubated with LPS (LPS), and melanocytes transduced with vector or BRAFV600E. The plot with the negative sample result also shows unstained WBCs (neg, dotted line). (**g**) Percentage of replicating CD3^+^ cells after induction with WBCs (neg), WBCs incubated with LPS (LPS), and vector or BRAFV600E-transduced melanocytes. Bars represent means ± standard deviation, n = 4. (**h**) Table of the five most enriched gene ontologies (FDR ≤ 5%) for genes that are up-regulated by RNA sequencing (FDR ≤ 5%) in *Tyr-NrasQ61K* over WT lymph nodes. (**i**) Column clustered heat map of all genes in the ontologies given in h for WT and *Tyr-NrasQ61K* lymph nodes. Genes are given by column and samples by row. The color intensity represents column *z-*score, where red indicates more highly expressed and blue indicates more lowly expressed genes. (**j**) Representative Foxp3 staining (brown) of albino WT and *Tyr-NrasQ61K* mice. Nuclei are counterstained with hematoxylin (blue). Scale bar = 50 μm. Quantification of the number of positive cells in two NRAS61K mice and two WT littermates, by calculating the average number positive cells from two slides per lymph node and counting three lymph nodes (two inguinal, one brachial) per mouse, showed no significant difference between the nodes (160.8 ± 21.1 vs. 174.3 ± 27.6, n = 2, *P* = 0.83). CSFE, Carboxyfluorescein diacetate succinimidyl ester; FDR, false discovery rate; ID, identification; LPS, lipopolysaccharide; neg, negative; WBC, white blood cell; WT, wild type.

Previously, a mouse model expressing activated NRASQ61K in melanocytes under control of a tyrosinase promoter (*Tyr-NrasQ61K*) was shown to be hyperpigmented because of an excess of melanocytes in the skin but also to contain melanocytes in the lymph nodes (Ackermann et al., 2005). Extending this observation in these mice, we showed that infiltration of pigmented melanocytes occurs into skin-draining (Figure 5a, inguinal and brachial) nodes but not into non–skin-draining nodes (Figure 5a, mesenteric) and spleen (Figure 5a, spleen). Although immunohistochemical analysis showed melanotic material dispersed throughout the skin-draining lymph nodes, particularly in older mice (Figure 5a), immunofluorescence analysis in lymph nodes of albino *Tyr-NrasQ61K* (but not wild-type) mice showed cells expressing the melanocyte marker DCT and exhibiting dendritic features characteristic of melanocytes predominantly localized adjacent to the subcapsular sinus of the lymph node (Figure 5b, Supplementary Figure S8a online). Therefore, to some extent, the melanotic material in the interior cortex of the node might reflect residual cell debris, including melanin (oxidized tyrosine polymers), after phagocytic digestion of melanocytes. To eliminate the possibility that localization of melanocytes to lymph nodes is unique to *Tyr-NrasQ61K* mice, we also confirmed in another mouse model of inducible BRAFV600E in melanocytes (*Tyr-Cre-Er:LSL-BrafV600E*) (Mercer et al., 2005) that 4 weeks after oncogene activation, predominantly in the skin of young adult mice (through topical application of tamoxifen), melanocytes also accumulated in skin-draining brachial and inguinal lymph nodes (see Supplementary Figure S8b).

As noted previously (Ackermann et al., 2005), compared with wild-type mice, the lymph nodes of *Tyr-NrasQ61K* mice were enlarged (Figure 5c, Supplementary Figure S8c), and their size progressively increased over at least the first several months after birth (Figure 5c). Moreover, the *Tyr-NrasQ61K* mice exhibited more melanocytes in the nodes than the *Tyr-Cre-Er:LSL-BrafV600E* mice (compare Figure 5a and Supplementary Figure S8b, *NrasQ61K* and *BrafV600E*), paralleling the relative numbers of melanocytes in the skin of each model (Ackermann et al., 2005, Dhomen et al., 2009).

To determine the proliferative status of the melanocytes in the lymph nodes, we used two-color immunofluorescence to stain melanocyte-containing lymph nodes of wild-type and *Tyr-NRasQ61K* albino mice for Ki67, a marker of cycling cells, and DCT, a marker of melanocytes. DCT-positive melanocytes in lymph nodes were invariably Ki67 negative, whereas large numbers of surrounding lymphocytes stained positive for Ki67 (Figure 5d). In fact, quantification of the cell fractions from the nodes, showed a massive expansion of nonmelanocyte (non–melanin-containing) cells (Figure 5e, left) and of CD3^+^ T cells in particular (Figure 5e, right), suggesting that the melanocytes present in the lymph nodes, directly or indirectly, induce a marked activation and expansion of T cells in these nodes. In sum, the increase in lymph node size appears to result primarily from expansion of T-cell populations in melanocyte-containing nodes.

T-cell activation and proliferation can be stimulated by MHC class II-mediated antigen presentation (Trombetta and Mellman, 2005). To investigate whether MHC class II-expressing melanocytes acquire the ability to activate T cells, we performed the mixed leukocyte reaction in which antigen-presenting cells stimulate proliferation of T cells. Indeed, BRAFV600E-expressing OIS melanocytes induced cell division in Carboxyfluorescein diacetate succinimidyl ester–labeled CD3^+^ cells in vitro, much more efficiently than control melanocytes (2.3-fold, *P* < 0.0001) (Figure 5f, 5g). Although this assay using unpurified T cells does not allow us to attribute T-cell activation to a direct physical interaction between T cells and MHC II on melanocytes, it does show that BRAFV600E mutant melanocytes are more able, either directly by physical interaction or indirectly via other cell types or secreted factors, to stimulate T cells than are control melanocytes.

To investigate whether melanocyte-containing lymph nodes of *Tyr-NRasQ61K* mice exhibited features of increased antigen presentation, we harvested RNA from lymph nodes and performed RNA-seq. This showed up- and down-regulation of approximately 577 and 423 genes, respectively (see Supplementary Figure S9 online). Remarkably, the top five gene ontologies represented in the up-regulated genes of *Tyr-NRasQ61K* nodes reflected increased antigen presentation and associated processes, such as endocytosis and vesicle-mediated transport (Figure 5h, 5i). In principle, T-cell expansion and antigen presentation in lymph nodes of *Tyr-NrasQ61K* mice could be associated with immune activation or induction of immune tolerance, the latter by activation of regulatory T cells (Delacher et al., 2014). To distinguish between these possibilities, we stained lymph nodes for expression of FoxP3, a transcription factor expressed by regulatory T cells (Delacher et al., 2014). This did not show an increase in the frequency of regulatory T cells in lymph nodes from *Tyr-NrasQ61K* mice (Figure 5j). We conclude that oncogene-expressing, nonproliferating primary melanocytes, directly or indirectly, facilitate an antigen presentation function and potential immune activation function in the lymph nodes.

To assess the significance of MHC class II expression in human melanocytic neoplasia, we mined human melanoma Cancer Genome Atlas data comparing expression of CIITA, HLA-DRA, and HLA-DRB with patient survival. Remarkably, high expression of each of these genes predicted improved patient survival (see Supplementary Figure S10 online). Moreover, HLA-DRA and HLA-DRB are both components of the recently defined “immune infiltration” gene expression signature that is associated with good prognosis in this disease (The Cancer Genome Atlas Network, 2015; data not shown). Of course, at least part of the CIITA, HLA-DRA, and HLA-DRB could be expressed by infiltrating immune cells themselves. However, previous studies have reported expression of HLA-DR on melanoma cells (Barbieri et al., 2011, Colloby et al., 1992, Pollack et al., 1981), and we also confirmed expression of HLA-DRA, HLA-DRB, and CIITA in a number of melanoma cell lines (Figure 3c). This underscores the importance of MHC class II expression in melanoma and is consistent with a tumor suppressor role for MHC class II.

## Discussion

Here we show that oncogene activation in primary human melanocytes is accompanied by up-regulation of MHC class II antigen presentation molecules, and phenotypes and functions suggestive of an antigen presentation role in vivo. Up-regulation of MHC class II molecules in OIS melanocytes is triggered by senescence-associated secretory phenotype factor IL-1ß, followed by IL-1ß–mediated up-regulation of CIITA, a master regulator of MHC class II expression (Trombetta and Mellman, 2005).

Several observations suggest that MHC class II expression in oncogene-expressing melanocytes has a dedicated function. First, melanocytes are restricted to the skin-draining nodes, suggesting that they reach the nodes specifically via the lymphatics, the normal route for migration of antigen-presenting dendritic cells, not nonspecifically via the blood. Second, these cells do not appear to be malignant, as judged by the absence of proliferation and their routine occurrence in mice lacking any detectable melanoma. Both the *NRas* and *Braf* models exhibit a long latency in progression to melanoma, of several months to more than a year (Ackermann et al., 2005, Dhomen et al., 2009). In both models, melanocytes were detected in nodes months before any melanoma was detected and is expected (e.g., at 39 days old). As noted previously, aggregates of apparently nonmalignant, nonproliferative, p16INK4a-expressing, melanocytic nevus-like cells, in the absence of any concurrent or subsequent melanoma, have also been reported in the skin-draining lymph nodes of humans (Mihic-Probst et al., 2003, Patterson, 2004) (albeit not as frequently or markedly as observed in the *Tyr-NrasQ61K* mouse model). Third, oncogene-expressing primary melanocytes appear to promote activation of the immune system. Oncogene-expressing senescent melanocytes stimulated T-cell proliferation in vitro in the mixed leukocyte reaction assay, and localization of melanocytes to the nodes was accompanied by a large increase in node-resident CD3^+^ T cells. RNA-seq analysis of lymph nodes also showed gene expression signatures characteristic of increased antigen presentation in the melanocyte-containing lymph nodes of *Tyr-NrasQ61K* mice. The increase in T cells was not accounted for by an overt increase in FoxP3-expressing tolerance-inducing regulatory T cells. In sum, although we cannot, of course, formally rule out the possibility that the node melanocytes are very early and/or failed micrometastases, the collective data from mice and humans at least suggest the possibility that oncogene-expressing premalignant melanocytes might be programmed to activate the adaptive immune system.

Other caveats should also be considered. In general, benign human nevi do not express MHC II, nor are they associated with immune infiltration (Campoli et al., 2012, Lyle et al., 2000). By IHC, we also found that nevi only very rarely (<10%) express detectable MHC II. Conceivably, benign human nevi represent a subset of OIS melanocytes that have been selected for down-regulation of MHC II via evasion of MHC II-mediated immune- editing. In apparent contrast to the data presented here, vemurafenib, a BRAFV600E inhibitor, was previously found to up-regulate IFN-mediated MHC II expression in A375 melanoma cells (Sapkota et al., 2013). A wider comparison of the functional relationship between BRAFV600E and expression of MHC II in transformed melanoma cells, compared with primary OIS melanocytes studied here, is justified.

Notwithstanding these caveats and the overall complexity of immune responses, composed of intricate temporally and spatially controlled antagonistic and synergistic interactions between many cell types, these data suggest a model whereby oncogene-expressing primary melanocytes up-regulate expression of MHC class II via an IL-1ß/CIITA autocrine loop. These melanocytes re-localize to the skin-draining lymph nodes, where they appear able to directly or indirectly stimulate proliferation of T cells. We propose that the ability of oncogene-expressing primary melanocytes to engage the adaptive immune system may facilitate tumor suppression.

## Materials and Methods

Details of materials and methods are available in the Supplementary Materials and Methods online (see also Supplementary Tables S5, S6).

### Cell culture

Lightly pigmented neonatal human epidermal melanocytes, human neonatal epidermal keratinocytes (both from Gibco, Waltham, MA) IMR90 fibroblasts (ATCC, Manassas, VA) were cultured according to supplier instructions. Infections with lentiviral vectors were performed as described (Pawlikowski et al., 2013). In all experiments, oncogene and control vector transduced cells were kept in culture under selection for 2 weeks before being assayed for senescence and gene expression. Alternatively, melanocytes were cultured in medium supplemented with 10 ng/ml recombinant growth factor listed in Figure 4e (all from Gibco, Waltham, MA) for 6 days.

### Genetically modified mouse strains

Mice carrying a tyrosinase promoter driven *NrasQ61K* gene (*Tyr-NrasQ61K*) have been described (Ackermann et al., 2005). Mice conditionally expressing the mutant *BrafV600E* gene under control of tyrosinase driven *CRE-ER* (Delmas et al., 2003) (*Tyr-CRE-ER:LSL-BrafV600E*) have also been described (Dhomen et al., 2009). Albino mice carrying the *Tyr-NrasQ61K* allele were generated by cross-breeding with the albino FVB/NJ (Taketo et al., 1991) strain. Control wild-type mice were littermate albino mice lacking the *Tyr-NRasQ61K* transgene. All experiments were carried out in compliance with UK Home Office guidelines at the Beatson Institute for Cancer Research (Home Office PCD 60/2607) under project license 60/4079.

### Microarray, RNA-seq, and The Cancer Genome Atlas data

Microarray and RNA-seq analysis of melanocytes transduced with BRAF600E expression or control vectors has been described (Pawlikowski et al., 2013); sequences are available from www.ncbi.nlm.nih.gov/geo (accession nos. GSE46818, GSE99397).

## Conflict of Interest

The authors state no conflict of interest.
