# Supplementary material for: Oncogene-Expressing Senescent Melanocytes Up-Regulate MHC Class II, a Candidate Melanoma Suppressor Function
Source: J Invest Dermatol. 2017 Oct;137(10):2197–207. doi: 10.1016/j.jid.2017.05.030 (PMC5613751; doi:10.1016/j.jid.2017.05.030)
Supplement: Supplementary Data [file mmc1.pdf]

SUPPLEMENTAL DATA

Table S1. Top 20 changes between BRAFV600E and control melanocytes, determined by Affymetrix array and RNAseq.

Affimetrix array[1]				RNAseq[2]		
Gene Symbol	UniGene ID	fold change	p (Holm)	Gene Symbol	locus	fold change
IL1B	Hs.126256	1798	5.80E-04	IL1B	chr2:113303654-113310988	28701
AREG	Hs.270833	1603	7.04E-03	C3	chr19:6628590-6671684	13965
HLA-DRA	Hs.520048	1384	6.57E-04	HLA-DRB1/5	chr6:32549180-32742436	6786
INHBA	Hs.583348	1289	5.58E-03	SERPINB2	chr18:59705918-59722158	6552
NPTX2	Hs.3281	1094	2.69E-03	AL121995.1/3	chr1:119778820-119903801	5922
TFPI2	Hs.438231	1087	9.02E-05	NPTX2	chr7:98099076-98099888	5333
TGFBI	Hs.369397	981	2.20E-04	HLA-DRA	chr6:32515610-32520802	5200
EREG	Hs.115263	886	3.95E-02	TNC	chr9:116821716-116920290	2761
HLA-DRB1/4	Hs.696211	883	1.21E-03	SAA1	chr11:18244360-18248092	2682
INHBA	Hs.583348	843	1.78E-03	TAGLN3	chr3:113200936-113215425	2289
C3	Hs.529053	772	2.95E-03	INHBA	chr7:41691160-41709216	1911
SERPINB2	Hs.594481	719	1.86E-02	TAC1	chr7:97199176-97207721	1885
TFPI2	Hs.438231	654	1.25E-02	HLA-DQB1	chr6:32549180-32742436	1762
COL22A1	Hs.117169	627	1.84E-03	MMP10	chr11:102146454-102156555	1690
CXCR4	Hs.593413	620	1.02E-01	TFPI2	chr7:93349289-93358297	1571
STC1	Hs.25590	613	9.92E-03	LCE1F	chr1:151017002-151017127	1560
ANGPTL4	Hs.9613	571	8.94E-02	MYH15	chr3:109646173-109646434	1402
RTN1	Hs.368626	568	8.39E-03	COL22A1	chr8:139707615-139995421	1327
STC1	Hs.25590	553	3.47E-04	EREG	chr4:75449369-75473334	1263
ANGPTL4	Hs.9613	571	8.94E-02	PRSS3	chr9:33740483-33789331	1244

BRAFV600E and control melanocytes were kept in culture under selection for 1 week before being assayed for senescence and gene expression as described (Pawlikowski et al., 2013), using either Affymetrix array (n=3) or RNA-seq (n=1). The full dataset can be obtained from www.ncbi.nlm.nih.gov/geo (accession no. GSE46818). See also Table S2 and S3 for Antigen presentation related transcript changes, detected by Affymetrix array and RNA-seq respectively.

[1]N=3 replicates. [2]N=1 replicate.

Table S2 . Antigen presentation related transcript changes between BRAFV600E and control melanocytes, determined by Affymetrix array.

Gene symbol	fold change[1,2]	p (Holm)
IL1ß	1797.5	0.001
HLA-DRA	1384.4	0.001
HLA-DRB1/4	883.2	0.001
CD74	250.8	0.016
HLA-DQB1	240.1	0.104
IL1A	237.8	0.002
BRAF	191.9	0.004
HLA-DRB1/3/4	182.9	0.018
IL8	158.0	0.134
IL11	130.7	0.016
HLA-DQA1	95.6	0.014
HLA-DMB	77.5	0.193
HLA-DRB4	46.2	0.256
IL33	32.2	1.000
HLA-DRB6	29.9	1.000
CTLA4	24.4	0.053
CIITA	22.6	0.111
HLA-DOA	15.8	0.107
HLA-DPA1	14.4	0.387
HLA-DMA	8.8	1.000
ICAM1	6.6	1.000
HLA-B	3.0	1.000
HLA-DPB1	3.0	1.000
HLA-DOB	1.8	1.000
CD28	1.8	1.000
HLA-F	1.4	1.000
HLA-C	1.4	0.302
HLA-E	1.3	1.000
HLA-DQB2	1.2	1.000
HLA-A	1.2	1.000
HLA-G	1.1	1.000
CD80	1.1	1.000
ICAM5	1.1	1.000
CD40	-1.1	1.000

[1]N=3. [2]Also see Table S3 for antigen presentation transcript changes detected by RNAseq. See Table S1 for additional information on the dataset.

Table S3. Antigen presentation related transcript changes between BRAFV600E infected and control melanocytes, determined by RNAseq.

Gene Symbol	Fold change[1,2]
IL1ß	28700.6
HLA-DRB1/5	6786.1
HLA-DRA	5199.7
HLA-DQB1	1762.4
HLA-DQA1	1013.4
HLA-DRB5	777.2
IL1A	771.9
HLA-DQA2	391.7
IL8	226.4
CD74	107.6
HLA-DMB	51.0
ICAM5	48.4
HLA-DOA	44.4
HLA-DOB	42.7
CD28	41.5
CIITA	31.7
CTLA4	21.3
HLA-DPA1	16.7
IL33	13.7
HLA-DMA	6.1
ICAM1	6.1
HLA-C	4.3
HLA-DPB1	3.2
HCG4P5,HLA-A	3.0
HLA-A/F	1.6
HLA-E	1.3
CD40	0.7
HHLA3	0.6
HLA-Z	0.5

[1]N=1. [2]Also see Table S2 for antigen presentation transcript changes detected by Affymetrix array. See Table S1 for additional information on the dataset.

Table S4. Mean fold change of cytokine quantities between the conditioned culture medium of BRAFV600E and vector transduced melanocytes.

Name	Fold change	P (Bh-fdr)[1]
IL1 beta	179.6	0.001
CCL7	131.8	0.001
CXCL5	100.1	0.000
CXCL1 alpha	64.0	0.000
VEGF	56.7	0.000
CCL5	33.0	0.035
CXCL1	10.8	0.000
IL8	8.3	0.000
IL6	4.2	0.008
GCSF	2.8	0.004
MCSF	1.9	0.490
CCL2	1.8	0.004
IL1 alpha	1.8	0.164
CCL8	1.6	0.617
IL7	1.6	0.459
INF gamma	1.3	0.620
IL2	1.3	0.557
CXCL9	1.2	0.396
IL12	1.1	0.593
IL3	1.1	0.798
CCL1	1.0	0.955
TGF beta1	1.0	0.955
ADAM11	1.0	0.836
TNF alpha	1.0	0.955
EGF	0.9	0.773
Angiogenin	0.9	0.121
IL13	0.9	0.891
CCL17	0.9	0.620
IL10	0.9	0.955
IL4	0.8	0.620
TNF beta	0.8	0.620
IL5	0.8	0.620
IGF1	0.8	0.955
Oncostatin M	0.8	0.463
PDGF BB	0.8	0.620
GM-CSF	0.8	0.754
SDF-1	0.7	0.955
CCL15	0.7	0.593
IL15	0.7	0.557
Leptin	0.5	0.251
Thrombopoietin	0.4	0.251
SCF	0.2	0.827

[1]Significance was calculated by students t-test with BH-fdr correction (n=4). Cytokine quantities in filtered culture supernatant from BRAFV600E and vector transduced melanocytes were determined using the human cytokine array G series 3 (Raybio). Culture supernatant was collected 2 weeks post transduction, and 2 days since the medium was last replaced.

Table S5. Quality control data of the RNA-seq of mouse WT and NrasQ61K lymph nodes.

Replicate ID[1]	Sample	Raw Sequence reads	Read Length	Aligned Reads (% of Raw Sequence reads)	Non-Duplicate Reads (% of Aligned Reads)
46419	wt	10,004,588	72PE	9,480,699 (94.76%)	8,186,445 (86.35%)
71309	wt	15,607,227	72PE	14,883,831 (95.36%)	13,392,117 (89.98%)
71310	wt	15,929,113	72PE	15,230,797 (95.62%)	12,793,310 (84.00%)
61600	*NrasQ61K*	13,785,721	72PE	12,808,334 (92.91%)	11,633,500 (90.83%)
68718	*NrasQ61K*	10,221,677	72PE	6,967,111 (68.16%)	5,621,199 (80.68%)
68722	*NrasA61K*	13,393,846	72PE	12,646,809 (94.42%)	10,159,113 (80.33%)

[1]The full dataset can be obtained from www.ncbi.nlm.nih.gov/geo, accession no. GSE99397.

Table S6. Primer sequences used for reverse transcription-real time PCR analysis.

Target	Forward primer[1]	Reverse primer[1]	probe[1]
hGAPDH	ACACCCACTCCTCCACCTTT	ATGAGGTCCACCACCCTGT	ATTGCCCTCAACGACCACTTTGTC
hACTB	AGAAGGATTCCTATGTGGGCG	CATGTCGTCCCAGTTGGTGAC	CTCACCCTGAAGTACCCCATCGAG
hCIITA	CACTAACCACGCTGGACCTT	GCAGAGCAAGATGTGGTTCA	CTTCTCCAGGCTGTATCCCATGAGC
hHLA-DRA	CCCAACGTCCTCATCTGTTT	AGCATCAAACTCCCAGTGCT	AAGTTCACCCCACCAGTGGTCAAT
hHLA-DRB	GCACAGAGCAAGATGCTGAG	GCAACCAGGTCCTGAGAAAG	TCCTGAGCTGAAATGCAGATGACC
hIL-1B	GCTGAGGAAGATGCTGGTTC	TCGTTATCCCATGTGTCGAA	TCCAGGAGAATGACCTGAGCACCTTC
mACTB	AGCCATGTACGTAGCCATCC	GCTGTGGTGGTGAAGCTGTA	CATCTACGAGGGCTATGCTCTCCCT
mH2-Ab1	CCTGGTGACTGCCATTACCT	ACGTACTCCTCCCGGTTGTA	TCGTGTACCAGTTCATGGGCGA

[1]All sequences are given 5’ to 3’.

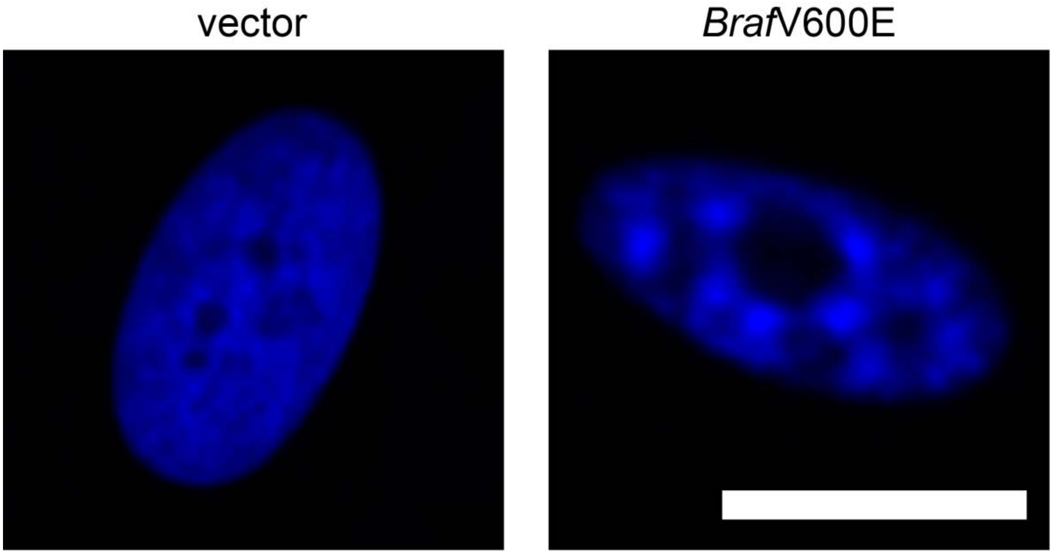

<u>Figure S1. Nuclei of senescent melanocytes display senescence-associated heterochromatin foci (SAHF).</u>
Confocal image of DAPI stained nuclei of vector control and *BRAF*V600E expressing melanocytes. Scale=10 µm.

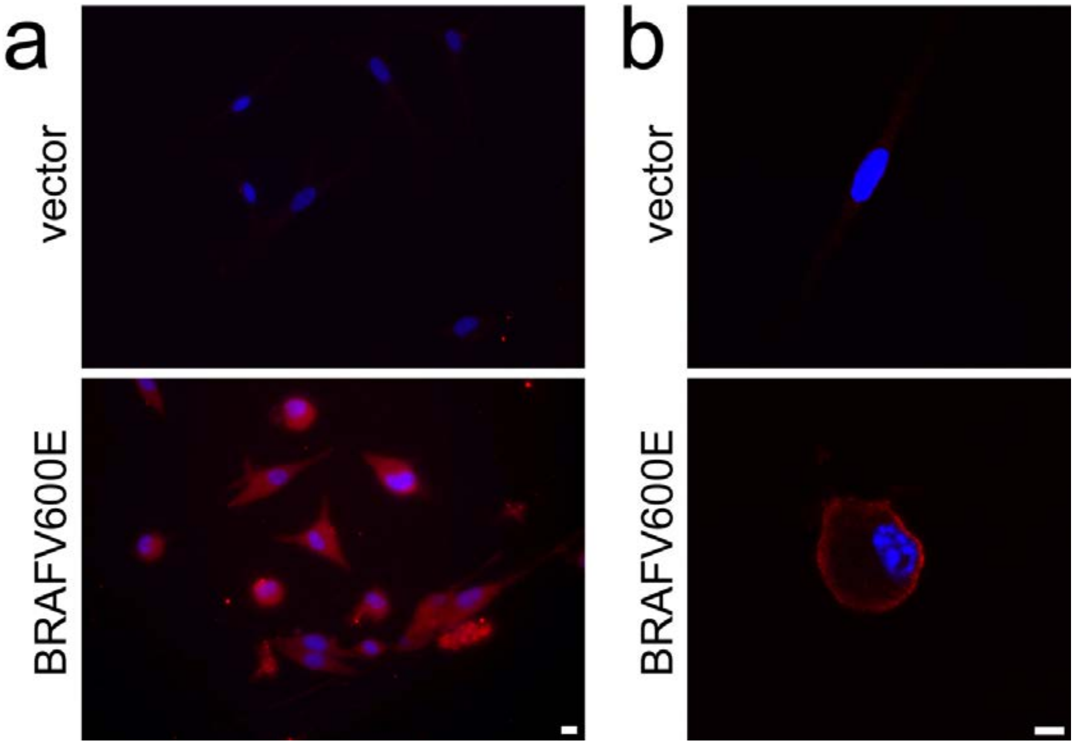

<u>Figure S2. Immunofluorescent microscopy of HLA-DR expression on BRAFV600E expressing melanocytes.</u>
A. Lower magnification overview IFM image of vector and BRAFV600E transduced melanocytes, showing DAPI stained nuclei in blue, and HLA-DR in red. Scale=100 µm.
B. Confocal IFM image of vector control and *BRAF*V600E expressing melanocytes, showing membrane localization of HLA-DR. Scale=100 µm.

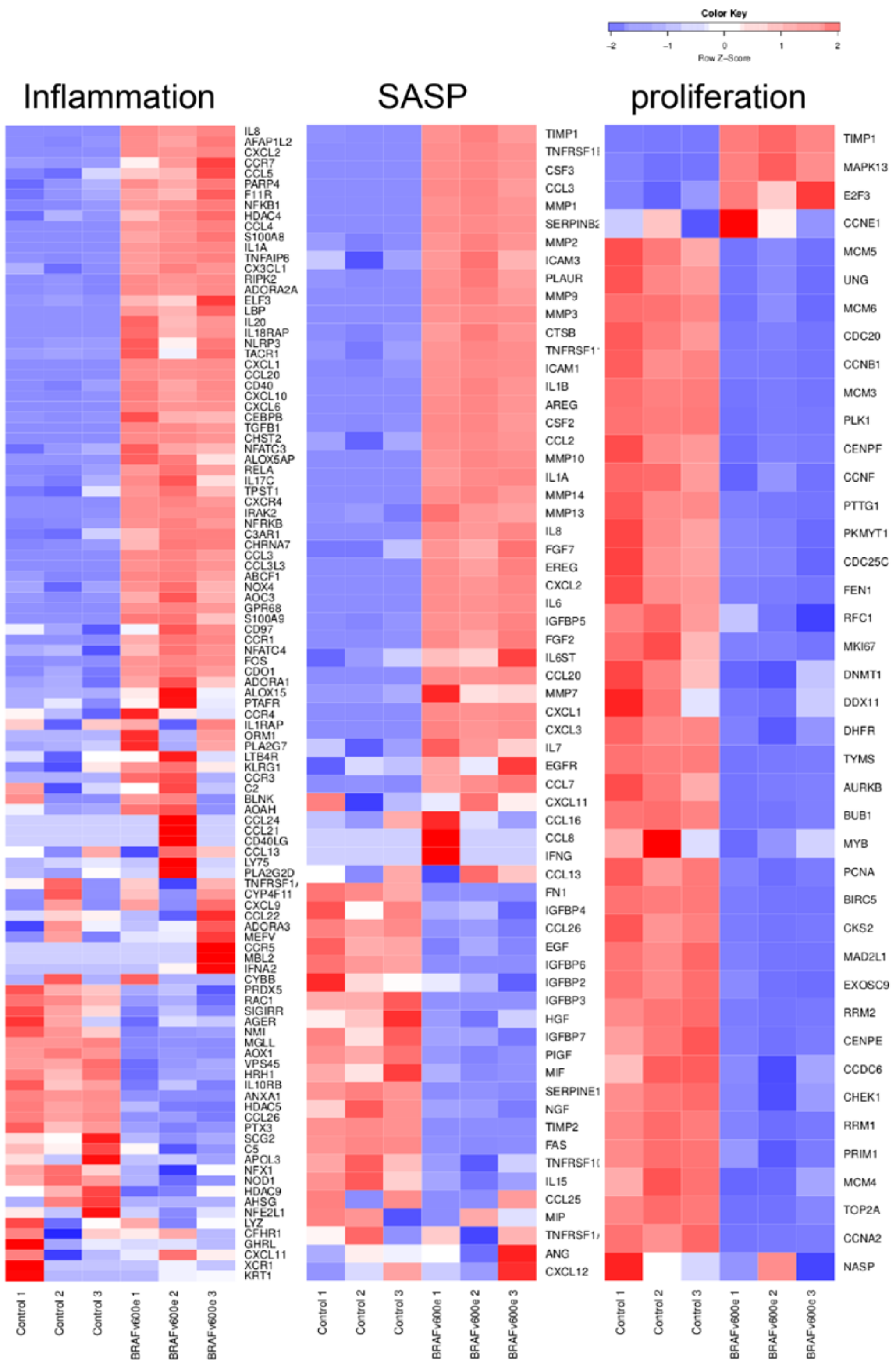

Figure S3. IMR90 fibroblasts expressing BRAFV600E display transcription profiles in compliance with senescence.

Correlation clustered heatmap of a curated list of known proliferation, inflammation and senescence associated secretory phenotype (SASP) genes. The colour intensity represents column Z-score, with red indicating high and blue low expression. The IMR90 fibroblasts were assayed 1 week after infection with BRAFV600E or control vectors.

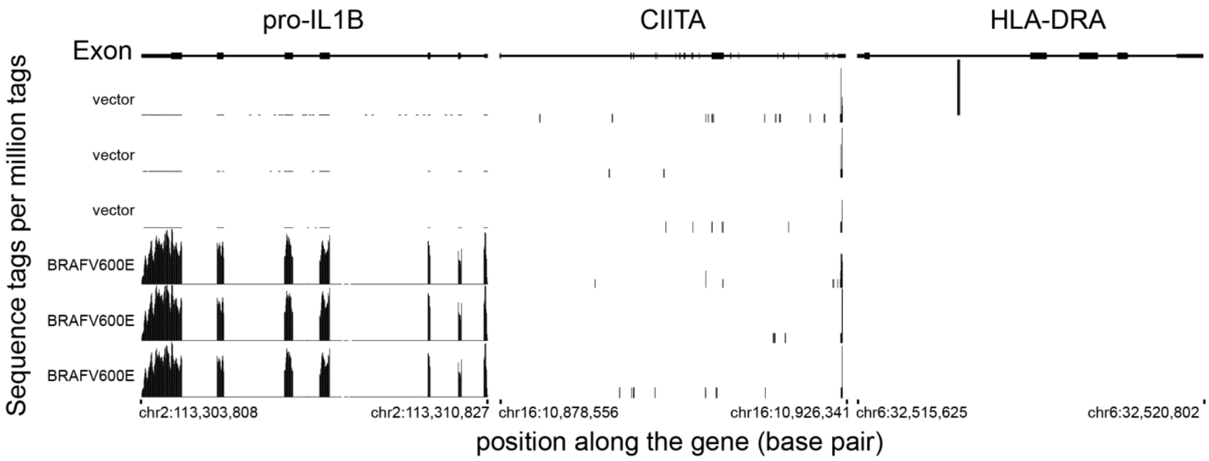

<u>Figure S4. IMR90 fibroblasts show IL1B, but not CIITA or HLA-DRA, transcripts upon BRAFV600E expression.</u>
Normalized RNAseq plots showing transcripts for the IL1B, CIITA and HLA-DRA genes, in IMR90 fibroblasts expressing either vector control or BRAFV600E. The IMR90 fibroblasts were assayed 1 week after infection with BRAFV600E or control vectors.

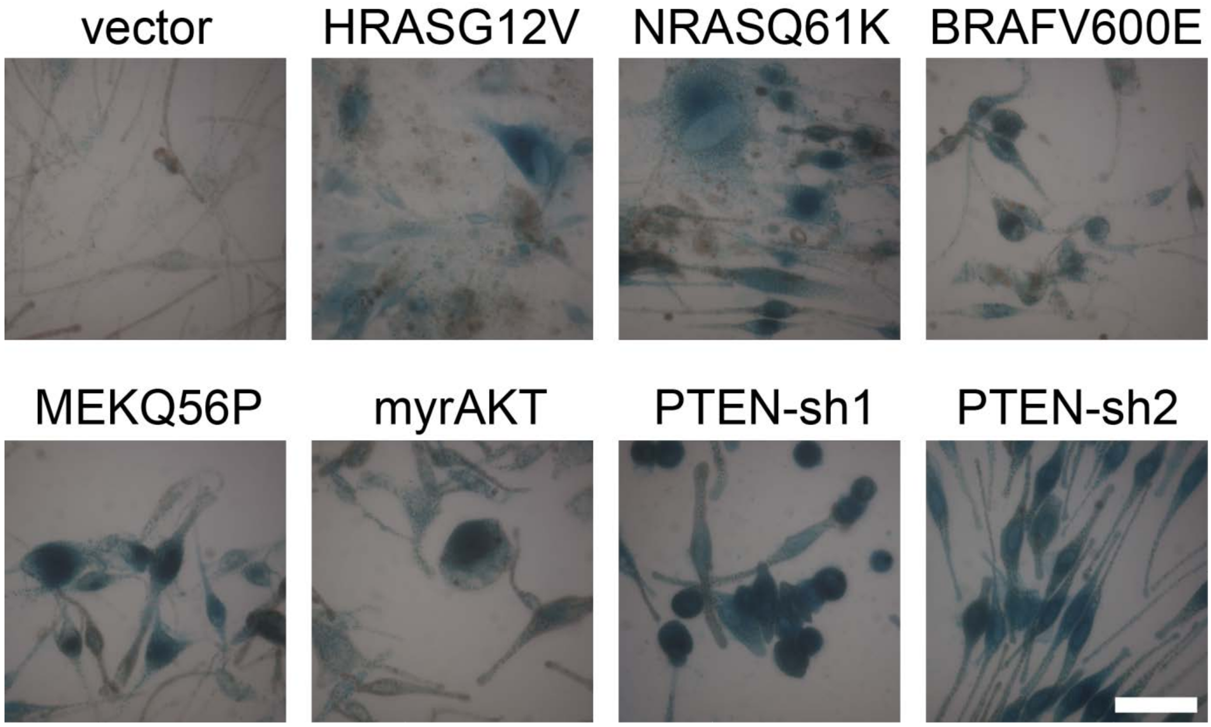

<u>Figure S5. Oncogene induced senescence in melanocytes is accompanied by expression of senescence associated beta-galactosidase expression.</u>
Representative staining for SA beta-galactosidase activity of melanocytes transduced with control vector, *HRAS*G12V, *NRAS*Q61K, *BRAF*V600E, *MEK*Q56P, myr*AKT* over-expression vectors and 2 different PTEN knockdown vectors for the quantified SA-Bgal counts shown in Figure 2G. Scale = 50 μm.

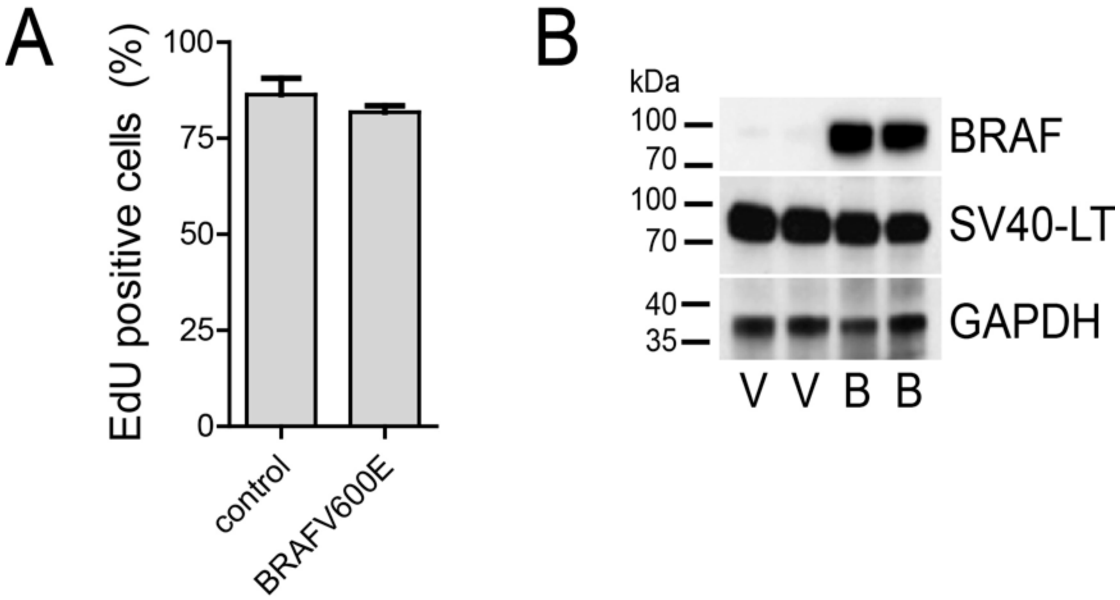

Figure S6. SV40 T-antigen can drive proliferation of melanocytes irrespective of mutant BRAFV600E presence.
A. Plot showing average percentage (n=3 +/- SD) of EdU positive melanocytes co-expressing SV40-T antigen and either vector (control) or BRAFV600E.
B. Representative Western blot showing expression of BRAF, SV40-LargeT and GAPDH as loading control.

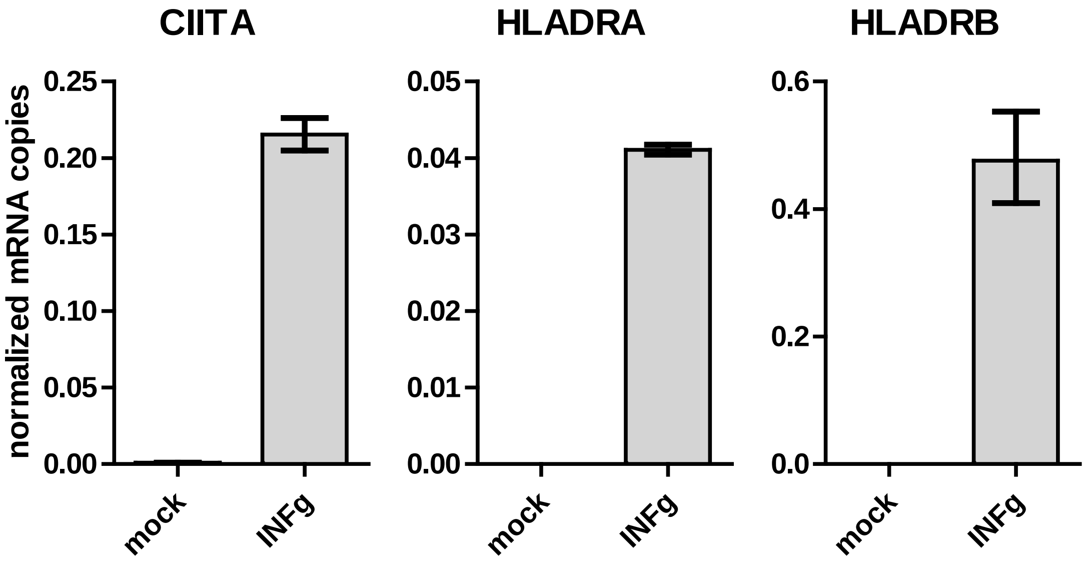

Figure S7. Interferon gamma induces CIITA and HLA-DR in melanocytes.
CIITA, HLA-DRA and HLA-DRB transcript levels detected by qRT-PCR analysis of mock or interferon gamma treated melanocytes. Graph depicts means +/- SD, n=4.

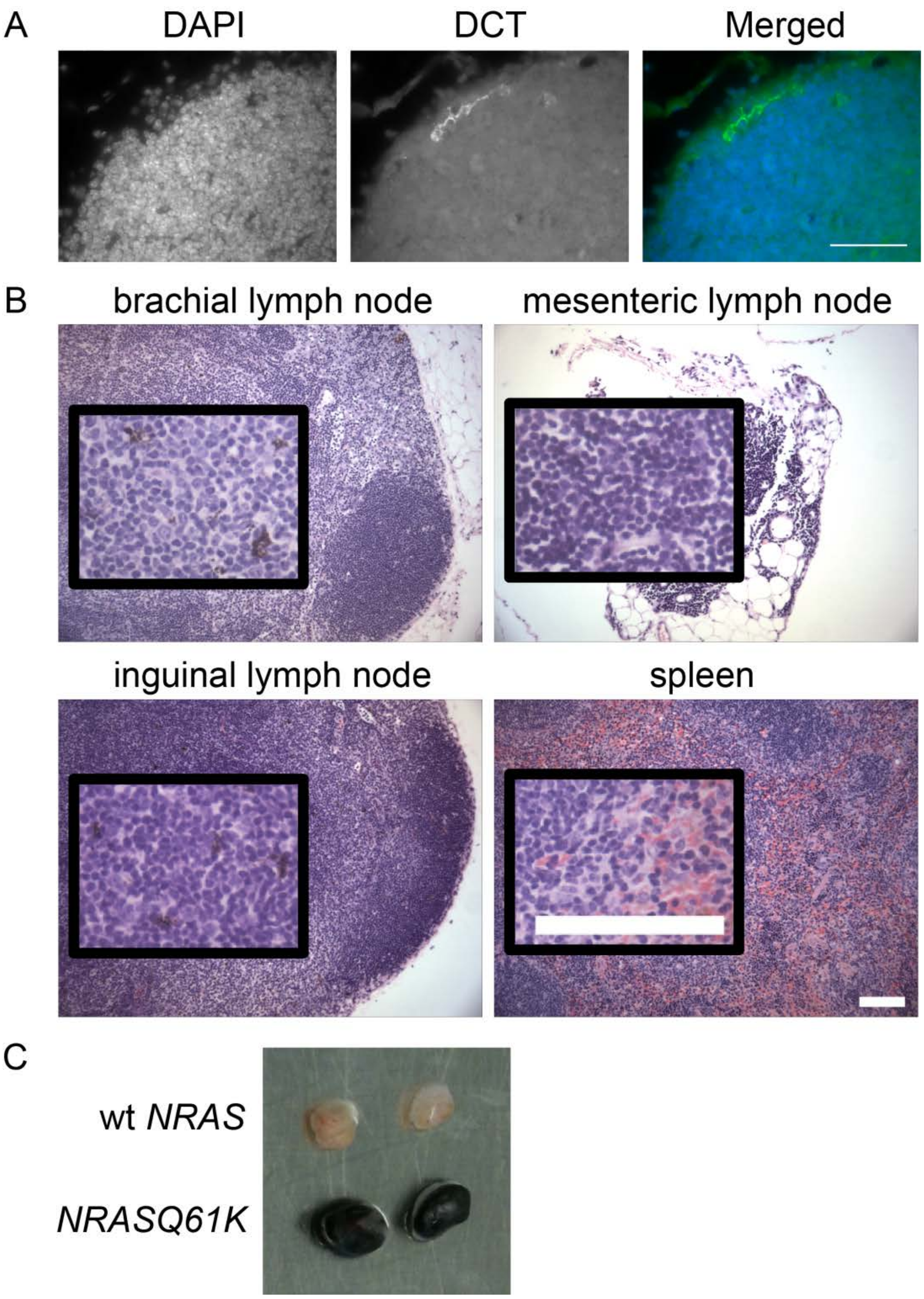

Figure S8. *Nras*Q61K and *Braf*V600E mutant melanocytes are present in skin draining lymph nodes.

A. Dendritic DCT (green)-expressing cells (presumptive melanocytes) adjacent to the subcapsular sinus of the lymph node of a Tyr-NrasQ61K mouse. DAPI, blue. Scale bar = 100 μm.

B. Haematoxylin and eosin stained sections of skin draining (inguinal and brachial) lymph nodes, non-skin draining (mesenteric) lymph nodes and spleen of Tyr-CRE-ER : LSL-BrafV600E mice. Note the pigment in the inguinal and brachial nodes. Scale bar = 100 μm.

C. Representative image of prepared inguinal lymph nodes from a WT mouse and a Tyr-NrasQ61K mouse, which expresses *NRAS*Q61K under the control of the tyrosinase promoter in melanocytes.

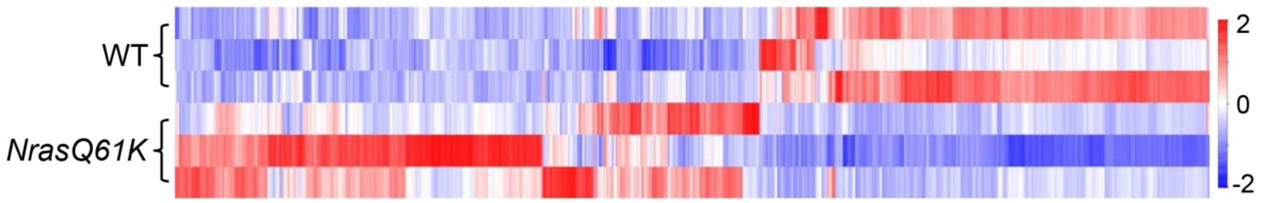

Figure S9. Differentially expressed genes between WT and *Tyr-NrasQ61K* lymph nodes.
Column clustered heatmap of differentially expressed genes (FDR <= 5%) between WT and *Tyr-NrasQ61K* lymph nodes. Genes are given by column and samples by row. The color intensity represents column Z-Score, where red indicates more highly expressed, and blue more lowly expressed genes. Heatmap shows up and down regulation of approximately 577 and 423 genes respectively. The full dataset can be obtained from www.ncbi.nlm.nih.gov/geo, accession no. GSE99397.

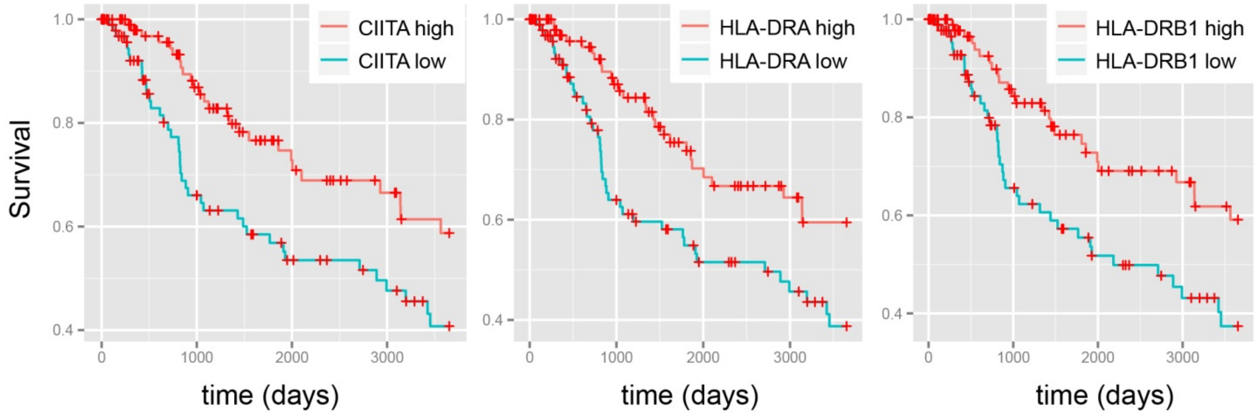

Figure S10. Skin cutaneous melanoma patient survival correlates with MHC II expression.
Kaplan Meier Curves of 10 year skin cutaneous melanoma patient survival (data from TCGA). Patients in the upper and lower quartiles of CIITA (left), HLA-DRA (middle) and HLA-DRB1 (right) expression are shown by the red and blue lines respectively ($p<0.001$, Cox Proportional Hazard Model). N=109 per quartile.

EXPANDED MATERIALS AND METHODS

Cell culture

Multiple batches of different lots of Lightly pigmented neonatal human epidermal melanocytes (Invitrogen) were cultured in medium 254 with human melanocyte growth supplement (HMGS), 100 U/ml penicillin and 100 µg/ml streptomycin (all from Invitrogen). Multiple batches of different lots of Human neonatal epidermal keratinocytes were cultured in EpiLife medium with human keratinocyte growth supplement, 100 U/ml penicillin and 100 µg/ml streptomycin (all from Invitrogen). Keratinocytes were cultured on collagen (Invitrogen) coated plates. IMR90 fibroblasts were obtained from ATCC and cultured in DMEM, supplemented with 20 % (v/v) fetal bovine serum, 100 U/ml penicillin and 100 µg/ml streptomycin (all from Invitrogen).

Lentivirus vector construction, production and infection

Lentivectors encoding CIITA, HRASG12V, NRASQ61K, BRAFV600E, MEK1Q56P, myrAKT, SV40 T-antigen or short hairpins directed against PTEN, CIITA and IL-1B, under the transcriptional control of the cytomegalovirus initial early promoter and puromycin or neomycin resistance from the simian virus 40 promoter were generated using standard methods; details available upon request. Vesicular stomatitis virus G pseudotyped lentivector stocks were produced as described previously (van Tuyn *et al.*, 2007). Melanocytes, keratinocytes and IMR90 fibroblasts were infected overnight in normal culture medium supplemented with 2 (melanocytes and keratinocytes) and 8 µg/ml polybrene, respectively overnight. Followed by 14-32 days of culture in the presence of 1 µg/ml puromycin or 250 µg/ml G418S (Invitrogen) to select for transduced cells.

In all experiments oncogene and control vector transduced cells were kept in culture under selection for 2 weeks before being assayed for senescence and gene expression as detailed below, unless stated otherwise.

Microarray, RNA-seq and analysis of TCGA data

Microarray and RNAseq analysis of melanocytes transduced with BRAF600E expression or control vectors has been described (Pawlikowski *et al.*, 2013), sequences can be obtained from the Gene Expression Omnibus (GEO) database, www.ncbi.nlm.nih.gov/geo (accession no. GSE46818, GSE99397).

Induction of melanocytes with conditioned medium

Cell culture supernatant was collected from BRAFV600E, vector or mock transduced melanocytes cultured in parallel at 2 weeks post transduction, and 2 days since the medium was last replaced (n=4 each). Vector transduced cultures were split several times to keep cultures at approximately the same number of cells. Culture supernatant was cleared by centrifugation at 3000 g for 10 minutes, followed by filtration through a 0.22 µm nitrocellulose membrane filter (Elkay), and frozen at -20°C until used in subsequent coculture experiments.

For the coculture experiments fresh melanocytes were cultured in parallel in a mixture of half normal culture medium as described above and half conditioned medium from either BRAFV600E, vector or mock transduced melanocytes (n=4 each). Medium was refreshed every 2 days, for a total of 2 weeks, at which time cells were harvested for RNA and RT-PCR analysis.

Induction of melanocytes with recombinant cytokines

Melanocytes were cultured in normal melanocyte growth medium (as described above), supplemented with 10 ng/ml of recombinant human Interleukin 1, beta (IL1B) (Gibco; PHC0816), recombinant human Vascular Endothelial Cell Growth Factor (VEGF) (Gibco; PHC9394), recombinant human Chemokine (C-X-C motif) ligand 1 (CXCL1) (Gibco; PHC1066), recombinant human RANTES (alternative name: CCL5) (Gibco; PHC1054), recombinant human Epithelial Neutrophil Activating Peptide-78 (ENA78, alternative name: CXCL5) (Gibco; PHC1336), or recombinant human Monocyte chemotactic protein 3 (MCP-3, alternative name: CCL7) (Gibco; PHC1574). Cells were cultured for 6 days in cytokine supplemented culture medium, which was refreshed every two days.

Genetically Modified Mouse strains

Animals were kept in conventional animal facilities and monitored frequently. All experiments were carried out in compliance with UK Home Office guidelines at the Beatson Institute for Cancer Research mouse facility (Home Office PCD 60/2607) under project license 60/4079. Mice were genotyped by PCR analysis. Mice carrying a tyrosinase promoter driven *NrasQ61K* gene (*Tyr-NrasQ61K*) have been described (Ackermann *et al.*, 2005). Mice conditionally expressing the mutant *BrafV600E* gene under control of tyrosinase driven *CRE-ER* (Delmas *et al.*, 2003) (*Tyr-CRE-ER : LSL-BrafV600E*) have also been described (Dhomen *et al.*, 2009). Albino mice carrying the *Tyr-NrasQ61K* allele were generated by cross-breeding with the albino FVB/NJ (Taketo *et al.*, 1991) strain. Control wild type mice were littermate albino mice lacking the *Tyr-NRasQ61K* transgene.

Reverse transcription-quantitative polymerase chain reaction analysis

Total RNA was isolated using the RNeasy mini spin kit with DNAse treatment (QIAGEN). Total cDNA was generated using SuperScript III (Invitrogen) from 0.1 - 5 µg RNA using random hexamers (Invitrogen), according to manufacturer instructions. Real-time qPCR was performed on $1/50^{th}$ of the cDNA, using the primers and FAM-labeled probes (IDT technologies) described in Supplemental Table S6. Reactions were performed on the Chromo4 PCR machine (Biorad), using platinum Taq, and dNTPs from Invitrogen. Transcript levels were quantified using standard curves of known quantities of plasmid DNA and normalized against the geometric mean of GAPDH and ß-actin (ACTB) gene transcripts.

Immunofluorescence microscopy

Cells were plated on glass coverslips and cultured at least 24 hours prior to fixation with 4% neutral buffered formaldehyde for 15 minutes at room temperature. Samples were washed 3 times with PBS, permeabilized with 0.1% triton in PBS for 5 minutes, followed by a further 3 washes with PBS. Cells were blocked for 30 minutes at ambient temperature with 4% bovine serum albumin and 0.02% sodium azide in PBS (blocking solution), followed by 1 to 24 hour labeling with anti HLA-DR (L243; Abcam) at 1 µg/ml in blocking solution at 4 °C. After 3 washes with PBS, cells were labeled with appropriate Alexa568 conjugated secondary antibodies (Invitrogen) in blocking solution for 1 hour at ambient temperature. Finally samples were washed 4 times with PBS and mounted in prolong gold with DAPI (Invitrogen). Images were acquired on the Nikon eclipse 80i fluorescent microscope, and the Olympus Fluoview 1000 IX81 confocal microscope.

Cytokine array

Culture supernatant from BRAFV600E and vector control transduced melanocytes was collected 2 weeks post transduction, and 2 days since the medium was last replaced. Culture supernatant was cleared by centrifugation at 3000 g for 10 minutes, followed by filtration

through a 0.22 µm nitrocellulose membrane filter (Elkay). Cytokine quantities were determined using the human cytokine array G series 3 (Raybio), according to manufacturer recommendations. Images were acquired on the Scanarray Express (Perkin Elmer).

ELISA

The human IL-1ß ESILA Kit from Thermo Scientific (EH2IL1ß) was used to measure the levels of IL1ß in the culture supernatants of Melanocytes transduced with BRAF600E or control vector following manufacturer instructions.

Immunohistochemistry

H&E staining and immunohistochemistry was performed as previously described (Pawlikowski *et al.*, 2013), using antibodies against DCT (Santa Cruz, sc-10451), FOXP3 (Abcam, ab54501) and Ki67 (Vector labs, VP-K451).

SA ß-gal and EdU assay

SA ß-gal staining was performed as previously described (Pawlikowski *et al.*, 2013). Staining for EdU incorporation was performed using the Click-iT EdU Alexa Fluor 594 Imaging kit (Invitrogen) according to manufacturer instructions, after a 72 hour pulse with EdU. Note that for all EdU experiments, a somewhat long 72 hour EdU pulse was used to truly be able to show a lack of proliferation in senescent cells, and to allow the generally slow proliferating primary cells (in the absence of BRAFV600E) used in this study to reach a significant percentage of EdU positive cells.

FACS

Fluorescence activated cell sorter (FACS) analysis was performed on a FACSCalibur system (Becton Dickinson), using standard methods. Where stated cells were stained with 5 µM CSFE or 1 µg/ml PI, or labeled with mouse anti CD3 conjugated to allophycocyanin (Biolegend).

Mixed Leukocyte Reaction

White blood cells were isolated according to standard protocols from excess human donor buffy coats using ficoll density gradient centrifugation, and labeled for 5 minutes with 5µM CSFE at room temperature. Unincorporated CSFE was removed by three washes with PBS. WBCs were plated at a density of $1x10^5$ cells per well in 96-wells conical wells (not-cell culture treated). Melanocytes previously transduced with BRAFV600E or control vector were added to $5x10^5$ cells per well. The co-cultures were maintained for 6 days in RPMI+10% FBS, medium was refreshed daily. FACS analysis was performed to assay WBC activity.

TCGA Data

The Cancer Genome Atlas (TCGA) skin cutaneous melanoma normalized RNA-seq V2 data was downloaded from TCGA data portal (http://cancergenome.nih.gov/).

Expression correlation network of TCGA skin cutaneous melanoma data

To generate the expression correlation network (ECN) from the TCGA skin cutaneous melanoma RNA-seq V2 data (Fig 3A), firstly a matrix of expression values by gene (n = 20,531) and patient (n = 375) was generated. Next for each pairwise combination of genes, the Pearson Correlation Coefficient (PCC) of expression values across all patients was calculated. Next, to reduce the number of potentially meaningless connections two filtering steps were applied: Firstly, correlations between two genes below 0.6 were filtered out. Secondly, for each gene, correlations equal to or above 0.6 were ranked (highest first).

Correlations that were not ranked amongst the top ten correlations of both genes were removed. Finally to generate the network, the resulting data was input into an equally weighted Fruchterman-Reingold force-directed algorithm, using a k value of 0.015 and 1000 iterations. Genes were set as nodes and correlations between two genes as edges.

RNA-seq of human Melanoma and Melanocyte cell lines

Paired-end reads were aligned to the human genome (hg19) using a splicing-aware aligner (tophat2) (Kim *et al.*, 2013). Reference splice junctions were provided by a reference transcriptome (Ensembl build 73), and novel splicing junctions determined by detecting reads that spanned exons that were not in the reference annotation. Bigwig files were generated from aligned reads using library size normalization, and uploaded to the UCSC genome browser (Kent *et al.*, 2002).

RNA-seq of mouse WT and *NrasQ61K* lymph nodes

Inguinal lymph nodes were prepared from 350 day old Tyr-NrasQ61K mice and WT littermates.
Total RNA was isolated using the RNeasy mini spin kit with DNAse treatment (QIAGEN). And prepared for RNAseq according to manufacturer instructions (Illumina) and as previously described (Pawlikowski *et al.*, 2013).
Paired-end reads were aligned to the mouse genome (mm10) using a splicing-aware aligner (tophat2) (Kim *et al.*, 2013). RNAseq quality and control metrics have been listed in Table S5. Reference splice junctions were provided by a reference transcriptome (Ensembl build 74), and novel splicing junctions determined by detecting reads that spanned exons that were not in the reference annotation. Aligned reads were processed to assemble transcript isoforms, and abundance was estimated using the maximum likelihood estimate function (cuffdiff) from which differential expression and splicing is derived (Trapnell *et al.*, 2013). Genes of significantly changing expression were defined as FDR corrected p-value <0.05.

RNA-seq Heatmaps

For each gene of biotype coding and status known in the reference transcriptome (Ensembl build 74) the FPKM value was calculated based on aligned reads, using Cufflinks (Trapnell *et al.*, 2013). Z-Scores were generated from FPKMs. Clustering was performed using the R library hclust2 and the Pearson method.

Gene Ontology analysis using David

Genes were uploaded to David (http://david.abcc.ncifcrf.gov), and a functional analysis performed using a background of Ensembl build 74 genes and the molecular functions GO terms.

Kaplan Meier Curve of 10 year skin cutaneous melanoma patient survival

The TCGA skin cutaneous melanoma normalized RNA-seq and patient clinical data was downloaded from the TCGA website (http://cancergenome.nih.gov/). Patients were filtered to include only those with both RNA-seq and clinical data, and a patient follow up date greater than 0. Patients were then grouped into quartiles by normalised CIITA, HLA-DRA and HLA-DRB1 expression level. Kaplan Meier Curves were plotted for the upper and lower quartiles of expression, using the R-library: survival (version 2.38-3). P-values were calculated using the function coxph.

Statistics

Unless otherwise specified significance was calculated using Student's t-test and graphs depict means +/- standard deviation.

SUPPLEMENTAL REFERENCES

Ackermann J, Frutschi M, Kaloulis K, McKee T, Trumpp A, Beermann F. Metastasizing melanoma formation caused by expression of activated N-RasQ61K on an INK4a-deficient background. Cancer Res 2005;65(10):4005-11.

Delmas V, Martinozzi S, Bourgeois Y, Holzenberger M, Larue L. Cre-mediated recombination in the skin melanocyte lineage. genesis 2003;36(2):73-80.

Dhomen N, Reis-Filho JS, da Rocha Dias S, Hayward R, Savage K, Delmas V, et al. Oncogenic Braf induces melanocyte senescence and melanoma in mice. Cancer Cell 2009;15(4):294-303.

Kent WJ, Sugnet CW, Furey TS, Roskin KM, Pringle TH, Zahler AM, et al. The Human Genome Browser at UCSC. Genome Research 2002;12(6):996-1006.

Kim D, Pertea G, Trapnell C, Pimentel H, Kelley R, Salzberg SL. TopHat2: accurate alignment of transcriptomes in the presence of insertions, deletions and gene fusions. Genome Biol 2013;14(4):R36.

Pawlikowski JS, McBryan T, van Tuyn J, Drotar ME, Hewitt RN, Maier AB, et al. Wnt signaling potentiates nevogenesis. Proc Natl Acad Sci U S A 2013;110(40):16009-14.

Taketo M, Schroeder AC, Mobraaten LE, Gunning KB, Hanten G, Fox RR, et al. FVB/N: an inbred mouse strain preferable for transgenic analyses. Proc Natl Acad Sci U S A 1991;88(6):2065-9.

Trapnell C, Hendrickson DG, Sauvageau M, Goff L, Rinn JL, Pachter L. Differential analysis of gene regulation at transcript resolution with RNA-seq. Nat Biotech 2013;31(1):46-53.

van Tuyn J, Pijnappels DA, de Vries AA, de Vries I, van der Velde-van Dijke I, Knaan-Shanzer S, et al. Fibroblasts from human postmyocardial infarction scars acquire properties of cardiomyocytes after transduction with a recombinant myocardin gene. FASEB J 2007;21(12):3369-79.
